# On the Pathology and Treatment of Varicocele

**Published:** 1851-11

**Authors:** 


*On the Pathology and Treatment of Varicocele.*—In a thesis with this title, M. Prunaire (*Gazette Medical de Strasbourg,*) speaks of a new mode of treating varicocele by caustic. He also enters minutely into certain anatomical considerations calculated to throw light on the pathology of the disease. In the first place, he has taken the measurements of the right and left spermatic veins in fourteen cases, together with the comparative weight of the testicles on either side. In classing the subjects according to their ages, he finds that between the ages of 3 months and 2 years, there was a difference of from one to two centimetres in favor of the left spermatic vein; between the ages of 30 and 35 years the difference was from two to three centimetres. As regards the size of the testicles, the author has constantly found the left larger and heavier than the right.

A recent writer has given the absence of valves in spermatic veins as the cause of varicocele, but this the author refuses to admit, as he concludes from the observations of St. Hilaire, as well as from his own researches, that valves are present in these veins in the majority of instances; this, of course, suffices to vitiate the theory arising out of their presumed absence.

The treatment adopted by M. Prunaire is thus described :—The pubes being denuded of hair, the patient is requested to walk for an hour or two; he is then chloroformed. An incision is next made over the cord, the sheath is opened with the utmost caution, and the veins scrupulously isolated from the other structures; this being done, some lint is passed underneath them to prevent the contact of the caustic with the subsequent tissues, and the veins are daubed with Vienna paste. After a few minutes the caustic is sedulously removed by washing the part in a dilute acid solution, and the wound is dressed. The veins which have been touched shrivel up, and when the eschar falls, are found to be completely obstructed.—*Prov. Med. and Sur. Journ.*

